# Supplementary figures and images for: Successful expansion and cryopreservation of human natural killer cell line NK-92 for clinical manufacturing
Source: PLoS One. 2024 Feb 23;19(2):e0294857. doi: 10.1371/journal.pone.0294857 (PMC10889882; doi:10.1371/journal.pone.0294857)

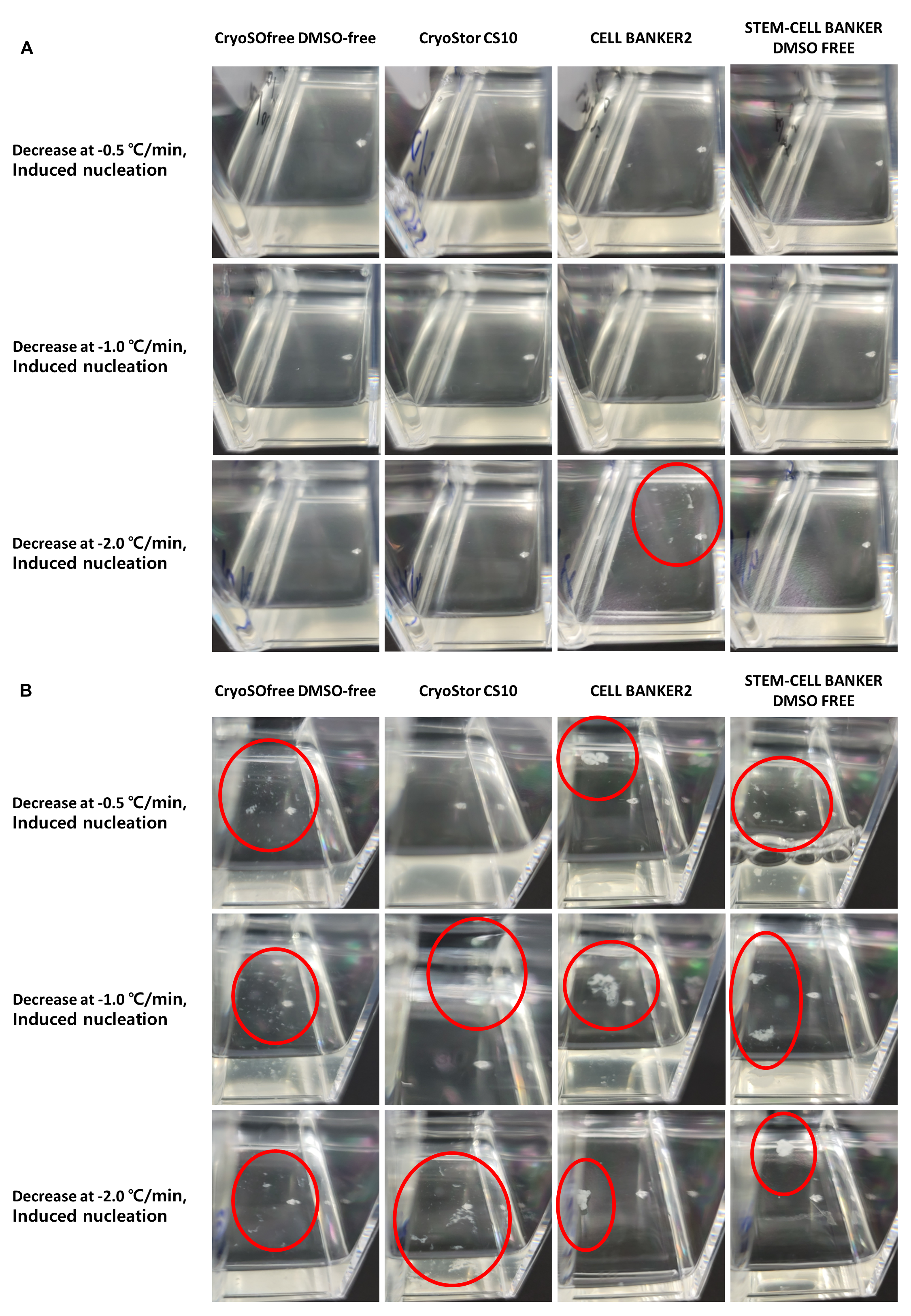

Supplement: S1 Fig — NK-92 cells were frozen using seven types of cryoprotective agents. After thawing the frozen cells, (A) Cultured in Xuri T-cell media after 24 hours. (B) Cultured in X-vivo 10 media to observe cell aggregation after 24 hours. (TIF) [file pone.0294857.s001.TIF]

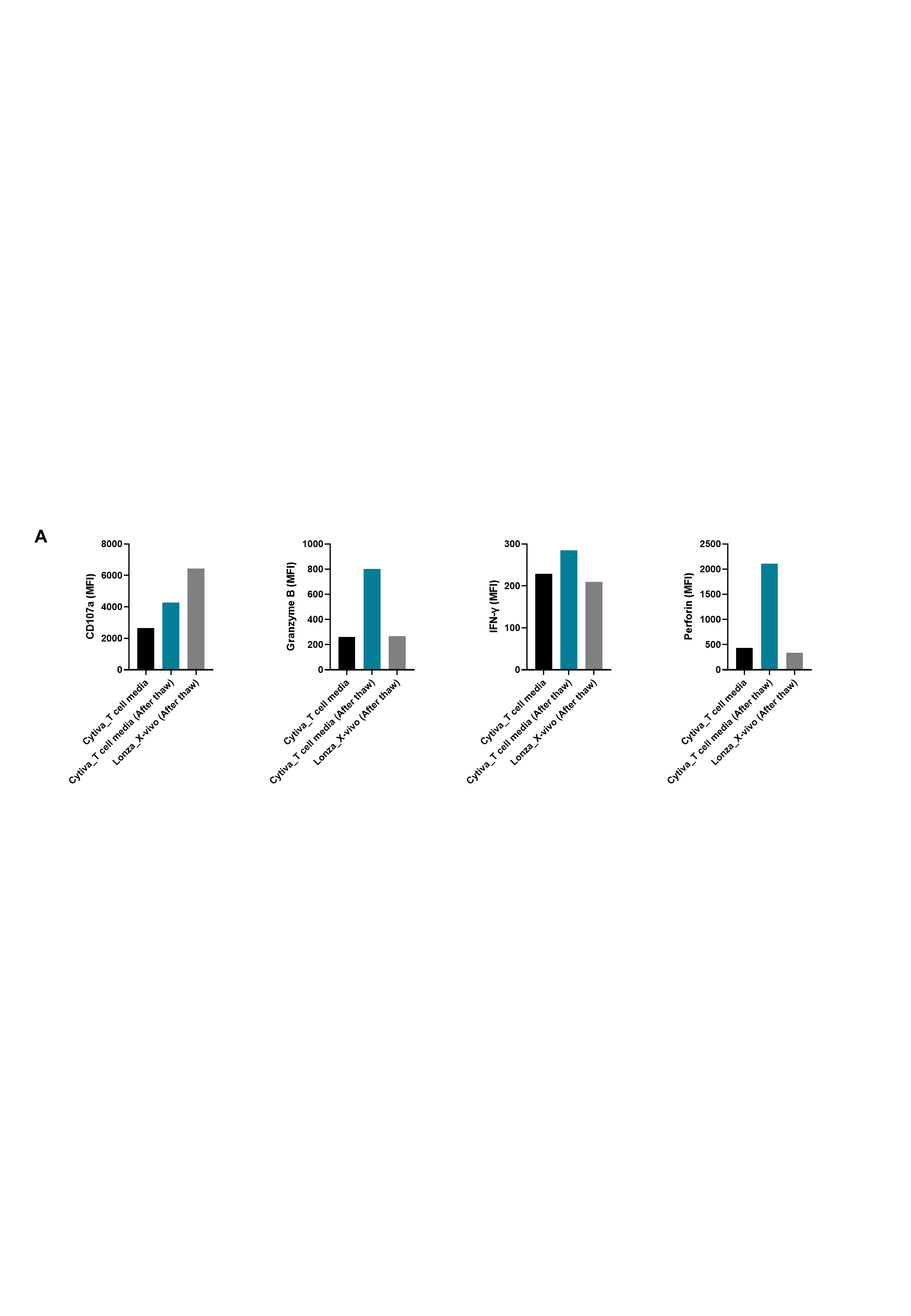

Supplement: S2 Fig — (A) NK-92 cells cultured in three distinct media were subjected to flow cytometry analysis to evaluate the expression of effector molecules. To quantify the alterations in expression levels, we represented the MFI ratios for each individual molecule as determined through flow cytometry measurements. (TIF) [file pone.0294857.s002.TIF]
